# Artificial Intelligence as a Core Public Health Competency: Proposal and Perspectives in Education and Research

**DOI:** 10.2196/92246

**Published:** 2026-09-30

**Authors:** Laura Brunelli, Federico Fonda, Silvio Brusaferro

**Affiliations:** 1 Department of Medicine, University of Udine, via Colugna 50, Udine, Friuli Venezia Giulia, 33100, Italy, 39 0432554768; 2 Accreditation, Quality and Clinical Risk Unit, Azienda Sanitaria Universitaria Friuli Centrale, Udine, Italy

**Keywords:** core competencies, public health, artificial intelligence, AI, education, research

## Abstract

The continuous development of AI presents unprecedented opportunities for public health. This development prompts educators and researchers to consider how AI can be applied to and integrated into a rapidly evolving global landscape in which AI is reshaping health systems. In turn, this generates an urgent need for structured proposals that bridge theory and practice. In this viewpoint, we present perspectives and proposals on AI applications in public health. We operationalize AI literacy as a core public health competency, defined through knowledge, skills, and attitudes and scaled across professional roles. For education, we describe how AI can support learners, teachers, and organizations through personalized learning experiences, dynamic scenarios, immersive simulations, content preparation, and communication strategies. For research, we analyze how AI can be embedded as a transversal enabler supporting the World Health Organization’s essential public health functions. We emphasize that AI should enhance, rather than replace, human capabilities and propose that AI literacy should be recognized as a core public health competency. We suggest that harnessing AI for public health education and research is, therefore, less a technological challenge than a collective responsibility shared by educators, researchers, and institutions.

## Introduction

The continuous development of AI technologies, defined as tools capable of imitating certain functions of human intelligence [1], prompts educators and researchers to consider how AI can be applied and integrated into public health education and research. AI is already being embedded in the daily work of public health, from outbreak detection to the design of health promotion campaigns [2], and growing evidence and debate on the promise of AI highlight the unprecedented opportunities these technologies offer, while also revealing significant challenges. In this context, educators and researchers navigate a complex landscape and have the opportunity to use AI technologies thoughtfully and strategically, aiming to realize their full potential while acknowledging their inherent limitations in both public health education and research. Addressing the implications of AI in public health education and research arises from the rapidly evolving global landscape in which AI is fundamentally reshaping health systems across diverse countries, with growing international implications. A potential gap between technological advancement and educational and research readiness [2-5] creates an urgent need for structured proposals at the international level that bridge theory and practice. This requires reflecting on perspectives and implications for public health education and research to ensure that current and future public health professionals are equipped with the technical, ethical, and critical thinking skills required to participate in and navigate these changes. Our central argument is that the response to this gap is not simply more technology, but a competency; AI literacy should be formally recognized as a core public health competency that is defined, taught, governed, and evaluated with the same rigor applied to other core disciplines such as epidemiology or health systems science [3,6]. The aim of this viewpoint is therefore threefold: (1) to define AI literacy as a core public health competency in terms of the knowledge, skills, and attitudes it comprises; (2) to examine how this competency can be developed in education and applied in research across learners, teachers, and organizations; and (3) to provide an appropriate framework for embedding and governing this competency at scale.

## Defining AI Literacy as a Core Public Health Competency

The concept of competency, following the competency-based education tradition, is defined as an observable ability that integrates knowledge, skills, attitudes, and values and can be developed and assessed along a continuum of expertise [7]. AI literacy is the ability to understand, use, monitor, and critically evaluate AI applications (including their limitations and ethical implications) within a given professional context or scenario [3]. AI literacy is related to, but broader than, general digital literacy; digital literacy concerns the use of digital tools and information, whereas AI literacy adds the capacity to reason about systems that are probabilistic, data dependent, opaque, and capable of generating plausible but incorrect outputs.

Operationally, AI literacy as a public health competency comprises three interdependent domains: (1) knowledge, (2) skills, and (3) attitudes. The knowledge domain includes the fundamentals of AI and machine learning, data literacy, and an understanding of the regulatory and ethical frameworks that govern AI in health. The skills domain includes the practical ability to interact directly or indirectly with AI systems (eg, formulating and iteratively refining prompts), to integrate AI outputs into public health tasks, and to critically appraise those outputs for accuracy, bias, and relevance. The attitudes domain includes ethical and reflective judgment, which involves recognizing when not to use AI, retaining accountability for decisions, and sustaining professional skepticism toward automated outputs. These domains develop along a continuum, from foundational awareness expected of all professionals, through applied competence for those who routinely use AI in practice, and to advanced expertise for those who design, validate, or govern AI systems. In fact, we argue that this competency should be tailored to the specific role(s) in the public health workforce.

We therefore distinguish 3 cumulative tiers that mirror the tiered logic of established public health workforce competency frameworks (eg, the Council on Linkages Core Competencies for Public Health Professionals, which separates frontline, program management, and senior leadership responsibilities [8]). At the operational level (user level; eg, frontline and program support roles), every professional and trainee should be a competent, critical user of AI tools in day-to-day work (eg, querying data, drafting and appraising health communication materials, and recognizing when an output is unreliable or inappropriate). At the applied level (service and research level; eg, program management and supervisory roles), those who select, adapt, implement, or evaluate AI applications need deeper methodological and data literacy (model validity, algorithmic bias, data provenance, and the appropriateness of a tool for a given population) so that they can judge not merely how to use AI, but whether and when to adopt it. At the strategic level (macro and stewardship level; eg, senior management and executive leadership roles), public health leaders and policymakers who set strategy for networks, regions, or national systems need competencies in governance, procurement, ethics, equity, and regulation to allocate resources, safeguard accountability and transparency, and steer system-level adoption in the public interest. These tiers are cumulative rather than mutually exclusive, as strategic leaders remain users.

Defining the competency also clarifies how its acquisition should be evaluated: per role and per scenario, with programs that should align assessment with established educational models (eg, Kirkpatrick levels, which distinguish reaction, learning, behavior, and results [9]). As a result, the field can move from teaching about AI to demonstrating changes in public health practice and, ultimately, in population health outcomes.

Acknowledging AI literacy as a core public health competency carries several implications, for both medical education and research. We discuss these implications in the following sections.

## Implications for Education

According to the United Nations Educational, Scientific and Cultural Organization (UNESCO), possible applications of AI in education include solutions designed for learners, teachers, and organizations. These applications may also involve support for the management and delivery of education, learning and assessment processes, enhancement of teaching methods, and continuing education [10]. In public health, the concept of “learners” extends beyond students enrolled in traditional academic programs to include health care professionals engaged in continuing medical education, as well as patients and communities. With the contribution of AI, learners may benefit from a tailored educational approach rather than a one-size-fits-all model, be supported in identifying personal learning gaps and needs, and experience personalized assistance [8-11]. For example, in public health preparedness, tools aimed at learners include those capable of a wide range of functions [4] as shown in Figure 1, such as the following:

- Generation of realistic and dynamic scenarios (ie, data-driven simulations and adaptive models with varying levels of complexity based on the learner characteristics)
- Training through immersive simulations (ie, serious games and virtual reality, interaction with AI agents for rehearsal)
- Predictive analysis and evaluation of decisions made (ie, automatic feedback, impact prediction supporting trial-and-error learning in a safe context)
- Personalization of the learning experience (ie, adaptive training based on the user’s level of knowledge and the preferences and needs of learners regarding the educational materials used)
- Analysis of individual and group performance (ie, simulation with multiple stakeholder perspectives and interaction)
- Support for interdisciplinary collaboration (ie, multiactor scenarios, supply chain, and logistics simulation)

**Figure 1.**
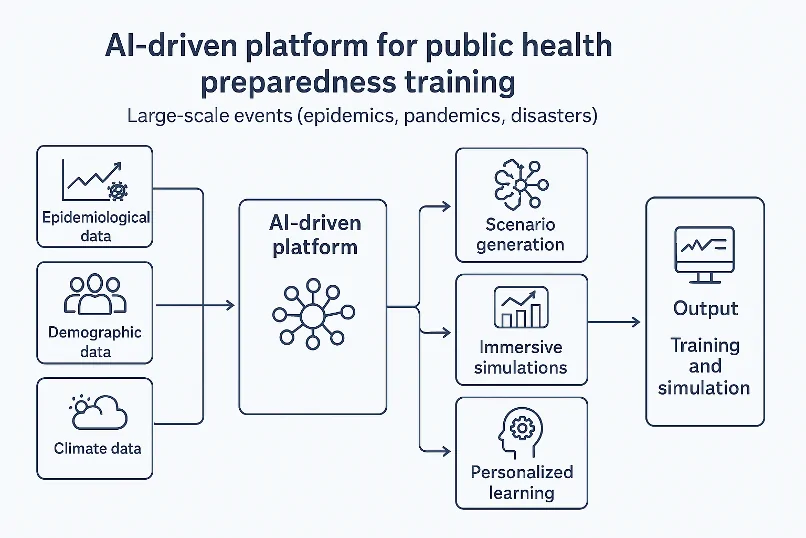
AI-driven platform for public health preparedness training: an example. Source: Image generated with OpenAI ChatGPT (GPT-5) and DALL·E, 2025.

Throughout this section we distinguish, in line with the competency defined above, between applications supported by established evidence, those with early or limited evidence, and those that remain speculative, since conflating the 3 types of applications risks overstating the maturity of the field. Adaptive, personalized learning and feedback are currently the best-evidenced applications; immersive simulations and virtual reality rehearsal are promising but supported mainly by early, short-term evidence, and predictive “impact scoring” of learner decisions remains largely speculative. Figure 1 provides an illustrative example of such integration. It depicts an AI-driven platform for public health preparedness training in which epidemiological, demographic, and environmental data inform the generation of dynamic outbreak scenarios, the delivery of immersive simulations, and the personalization of individual learning pathways, with learner performance subsequently feeding back into further training. In doing so, the figure operationalizes the operational (user) tier of the competency within a realistic preparedness setting for large-scale events and illustrates how otherwise discrete learner-facing functions can be coordinated into a single, coherent training system rather than deployed in isolation. For example, a concrete learning goal at this tier is that every public health trainee should be able to use a generative AI tool to draft and then critically correct a piece of health communication content, identifying errors and biases.

Similarly to learners, the concept of “teachers” in public health extends beyond traditional academic faculties to include public health practitioners and professionals involved in primary prevention, health promotion, and community-based education. In this context, AI can support teachers and public health educators across all settings, from academia to primary prevention and health promotion. For example, AI could assist with information selection and visual representation when preparing teaching materials, help to better understand learners’ learning processes and outcomes to inform decisions on educational strategies and interventions, perform monotonous tasks such as marking exams, and provide simple feedback about lesson and exam schedules [12,13]. AI can also help teachers and organizations effectively communicate health promotion and prevention content, adapting it to the abilities and characteristics of the target population, and considering linguistic [5], cultural, literacy, and health literacy differences [14], as well as any disabilities [4]. Following an optimistic vision of AI implementation for public health [15], when used for good, AI could also help counteract infodemics (defined by the World Health Organization [WHO] as an overabundance of information, including false or misleading information, during a health emergency). AI-based screening methods could be used to check online big data for fake news, with machine learning or artificial neural networks searching social media for recurrent words, phrases, images, or other problematic patterns and flagging content automatically for human review, rather than replacing editorial and public health judgment [16].

Organizations have a transversal role, bridging learners and teachers and supporting public health education at multiple levels. They can facilitate and provide access to AI technologies for both groups, promoting effective teaching and learning. Organizations may also benefit from AI in administrative tasks, such as enrollment forecasting, resource allocation optimization, and automation of school operations [4]. Moreover, organizations are called to act as stewards of responsible AI use, establishing policies, monitoring implementation, and ensuring that AI applications align with ethical and legal standards, thereby safeguarding collective educational processes and public health delivery. In practice, this stewardship role means issuing acceptable use and transparency guidance for teachers and students, investing in faculty development so that teachers themselves reach applied competence, defining assessment safeguards that preserve academic integrity, creating governance structures with clear accountability, and addressing inequities in access to tools and infrastructure.

## Implications for Research

In public health research, the systematic use of big data, the Internet of Things, and Industry 4.0 products is already becoming part of everyday life for researchers, with AI applications being developed in an increasingly wide range of areas (eg, development of new drugs and therapies, identification of biomarkers for population screening, identification and prediction of individual and community risk factors, etc). AI software already enables studies that can personalize responses by integrating knowledge derived from population data (real or simulated), the exposome, and “omic” sciences [15,17,18]. Moreover, AI can support researchers in project management and data acquisition, such as using augmented reality tools (eg, portable devices [19]), social listening, and data mining to find patterns, trends, correlations, anomalies, and features in datasets [16] or on social media [2]. This can aid behavioral epidemiology and deep learning for the One Health approach. AI support extends through to data analysis (eg, augmented reality tools, satellite image analysis for environmental hygiene, medicine in conflict zones or natural disasters), and the identification, implementation, and scientific validation of possible responses.

We suggest that a structural priority for public health research is to assess how, and to what extent, AI can be integrated into public health systems in relation to its contribution to the essential public health functions (EPHFs) of the WHO [6]. Framing AI as an enabling tool for the EPHFs provides a system-level analytical framework, allowing researchers to link technological innovation to surveillance, preparedness, prevention, health promotion, governance, workforce development, research, and equity. When strategically implemented and empirically evaluated, AI has the potential to support several, if not all, of the 12 EPHFs. While AI is not a standalone solution, it can act as a transversal enabler that strengthens public health capacities across the entire policy and service continuum (Table 1). In fact, the EPHFs are the set of fundamental activities (spanning surveillance, emergency management, stewardship, financing, health protection, disease prevention, health promotion, community engagement, workforce development, quality and equity, research, and access to health products [6]) that every health system must perform to protect and improve population health. The WHO defined them precisely to give countries a common, globally legitimate reference for assessing and strengthening public health capacity. Because they are already used for workforce planning and are explicitly cross-sectoral, they allow innovation to be linked to system-level needs rather than to isolated tools. Framing AI as a transversal enabler of the EPHFs therefore gives researchers an analytical lens for prioritizing and evaluating AI against population health functions rather than technological novelty. Moreover, because one of these functions is workforce development (EPHF 9), the same map that shows where AI can strengthen surveillance or prevention also identifies the competencies the workforce must acquire to deliver those functions responsibly, tying the research agenda directly to education.

**Table 1.** Essential public health functions (EPHFs): examples of AI applications and main associated challenges.

Essential public health function	Examples of AI application	Main associated challenges^a^
EPHF 1: Public health surveillance and monitoring	Support for data surveillance and monitoring, including natural language processing for interpreting medical records and social media/web searches/contents monitoring to detect early outbreak signals (social listening)	Privacy and data protection
EPHF 2: Public health emergency and management	Generation of dynamic interpretative scenarios to support public health emergency management decisions, predictive analytics	Overreliance on automated predictions
EPHF 3: Public health stewardship	Automation of administrative operations, support complex decision-making	Unclear accountability and responsibility for decisions
EPHF 4: Multisectoral planning, financing, and management for public health	Optimization of allocation of financial and human resources for resource forecasting	Algorithmic bias in resource allocation
EPHF 5: Health protection	Environmental hygiene, forecasting environmental risks	Data quality and representativeness
EPHF 6: Disease prevention and early detection	Accelerate the identification and prediction of individual and community risk factors	Algorithmic bias and risk of stigmatization
EPHF 7: Health promotion	Chatbots to provide real-time information (eg, healthy lifestyles, symptoms, and vaccines) and personalization of health messages, helping to contrast infodemics with fake news screening	Accuracy of automated outputs and misinformation
EPHF 8: Community engagement and social participation	Facilitation for understanding complex medical language, customization of messages and adaptation for communities for greater inclusivity	Digital exclusion and inequitable access
EPHF 9: Public health workforce development	Personalized training for professionals, assisted identification of individual learning gaps, immersive simulations and scenarios	De-skilling and overreliance on automated systems
EPHF 10: Health service quality and equity	Assisted technologies to ensure quality and equity to all populations, targeting individual needs	Equity gaps and bias
EPHF 11: Public health research, evaluation, and knowledge	Support in data acquisition and complex analysis, accelerate the development and implementation of quality evaluation projects	Transparency and reproducibility (“black box”)
EPHF 12: Access and utilization of health products, supplies, equipment, and technologies	Assisted utilization of complex technological infrastructures, assisted supply chain and forecasting of population needs	Data interoperability and accountability

a The challenges listed are illustrative and not exhaustive; most are cross-cutting and apply to varying degrees, across all functions. Notable cross-cutting challenges include privacy and data protection, algorithmic bias and discrimination/stigmatization, lack of transparency and explainability (black box), unclear accountability, overreliance on automated systems, digital exclusion and inequitable access, misuse or misinterpretation of predictive outputs, and the overarching need for ethical governance.

## Issues and Ethical Implications

We identify several unresolved issues. In research, it is not yet clear to what extent AI can, or should, be credited as a coauthor of publications [20], or act as a semiautonomous investigator (eg, in systematic reviews [21]) especially when population data are involved. Beyond authorship, the main challenges include the overall protection of data (security, quality, accuracy, availability, and privacy); the environmental sustainability of systematic AI use, given its energy and carbon footprint [22-24]; the social and professional impact of these technologies (possible inequalities arising from uneven access to and use of digital tools [14,25]), the dehumanization and intrusiveness of AI-mediated teaching and learning [10], and the de-skilling of professionals, especially in the early stages of their careers [26]; and the potential for malicious use through misinformation and disinformation. In addition to these, there are risks inherent in the technology itself: AI is prone to hallucination and inaccuracy (returning plausible but false information), is not free from algorithmic bias (arising from unrepresentative data or design choices), and often behaves as a “black box” whose reasoning cannot be fully traced. A subtler issue is the information asymmetry emerging between generations, which can invert the usual direction of expertise; handled constructively, it becomes an opportunity for teachers and more experienced colleagues to manage uncertainty together with younger ones [26].

These concerns could become clearer, and easier to act on, when organized into four categories: (1) governance, (2) equity, (3) pedagogical integrity, and (4) professional practice, whose weight differs across the research and education domains. In research, governance is chiefly about authorship, autonomous review agents, and the lawful use of data; in education, it is about acceptable use policies, assessment integrity, and transparency regarding when and how AI has been used. This governance should be anchored in the growing body of responsible AI guidance, from the WHO’s work on the ethics and governance of AI for health to academic frameworks such as that of Char and colleagues [27] on implementing machine learning in health care. Equity, in turn, is sharpened by well-documented algorithmic bias; previous research has shown, for example, that a widely used clinical algorithm underestimated the needs of certain patient groups because it used cost as a proxy for illness [28], a risk to both research validity and the fairness of any service or educational tool built on biased data. Pedagogical integrity (dehumanization, overreliance, de-skilling) is chiefly an education domain concern, whereas professional practice risks, such as misinformation and AI’s energy and carbon footprint, span both. Seen this way, the information asymmetry noted above is not merely a difficulty to be endured but part of the competency: managing it transparently and ethically is a professional skill to be taught.

## Conclusions and Future Directions

AI application in both education and research in public health can follow two strategic approaches, as reported by Abdulnour and colleagues [26]: (1) the centaur approach, in which the user strategically divides tasks between themselves and the AI, allocating responsibilities according to the strengths and capabilities of each; or (2) the cyborg approach, where the user works with AI iteratively by prompting, correcting, and requesting justifications, then refining the output jointly with the AI. Regardless of the chosen approach, as stated in the UNESCO guidelines for policymakers, we emphasize that AI should be used to enhance, rather than replace, human capabilities, shifting the paradigm from AI to augmented intelligence [10], where human skills are empowered by the specific capabilities provided by up-to-date technologies. All these developments and implementations must consider the regulatory framework surrounding AI at both international and national levels. WHO documents have outlined the main principles to be followed (ie, “Ethics and Governance of Artificial Intelligence for Health” [29]) and have further analyzed large language models [30] and benchmarking among AI systems supporting health care [31]. For example, the same discussion has taken place at the European level, with the release of the AI Act [32], and at the Italian national level as well [33]. In addition, guidelines for the evaluation of studies conducted with the aid of AI have been published [34]. The final goal should not be to replace public health professionals, teachers, or researchers, but to provide an additional tool for professionals who are already trained and capable of identifying the problem, formulating the research or teaching question, categorizing the data, developing the algorithm, deciding how the pieces of the puzzle fit together, and drawing conclusions based on the data [10]. In fact, according to the Moravec paradox, even if AI may be better at pattern discovery and statistical reasoning, it is poor at self-directed learning, applying common sense, and making value judgments [35]. This principle of augmentation has a precedent worth naming. Friedman’s [36] “fundamental theorem” of biomedical informatics holds that a person working in partnership with an information resource should perform better than that person unaided. We propose an analogous fundamental principle for AI in public health: an AI system is valuable only insofar as a competent public health professional supported by it achieves better population health outcomes than the same professional working without it.

The pace and shape of responsible AI governance are not uniform across the world, and the reasons are structural rather than incidental. Where health systems are largely publicly funded and health is treated as a public good, the surrounding incentives tend to favor precaution, equity, and shared standards, and governance often seeks to guide innovation from the outset. Where the development and deployment of AI are driven more strongly by market competition, governance tends to evolve alongside (and at times behind) innovation, reflecting different priorities such as speed, scalability, and user choice. Keeping in mind that public health is inherently public good, each orientation carries its own risks, from overcaution and delayed adoption on one side to fragmentation and uneven safeguards on the other. The challenge is to find the best possible balance between values and constraints.

We therefore suggest several priority actions in public health. Educators should define AI literacy learning outcomes for each learner group and assess them beyond satisfaction, using established models, and embed critical appraisal exercises that make the limitations of AI explicit. Institutions and organizations should issue acceptable use and transparency guidance, invest in faculty development, protect assessment integrity, and address inequities in access. Researchers should prioritize studies that evaluate AI’s real-world contribution to specific EPHFs, with explicit attention to bias and equity, and develop the governance and reporting standards needed for AI’s evolving role in the research process itself. Most importantly, we believe that it is important to provide cross-disciplinary, continuous, and systematic AI training for students, postgraduates, health care professionals, and researchers to improve individual levels of AI literacy [26,37], embedding it as a core public health competency, comparable to epidemiological methods or health systems knowledge [6]. Such training should include fundamentals of AI and machine learning, data literacy, prompt engineering and interaction with generative AI, limitations and risks of AI, practical applications in public health, ethics, transparency, governance, practical skills, and tools. It is also essential to promote and develop research in various fields of application, test its impact and real effectiveness in practice, and govern its implementation in traditional health care sectors including health promotion, disease protection, and prevention, with a view to personalization for individuals and communities. At the end, we suggest that harnessing AI for public health education and research is, therefore, less a technological challenge than a collective responsibility shared by educators, researchers, and institutions. In the near future, the question will no longer be whether AI will shape public health practice, but how to engage with it critically, ethically, and competently. Ultimately, the value of AI in public health will be determined not by the advancements and sophistication of algorithms, but by the wisdom with which humans choose to deploy them.

## Abbreviations

EPHF: essential public health function
UNESCO: United Nations Educational, Scientific and Cultural Organization
WHO: World Health Organization

## References

[1] World Commission on the Ethics of the Scientific Knowledge and Technology (2019). Preliminary study on the ethics of artificial intelligence. https://unesdoc.unesco.org/ark:/48223/pf0000367823.

[2] Panteli D, Adib K, Buttigieg S (2025). Artificial intelligence in public health: promises, challenges, and an agenda for policy makers and public health institutions. Lancet Public Health.

[3] Semi MMA, Das S, Utsho MR (2026). Artificial intelligence in public health education: a scoping review of workforce competency development. Health Sci Rep.

[4] Wang J, Li J (2024). Artificial intelligence empowering public health education: prospects and challenges. Front Public Health.

[5] Naumova EN (2024). Artificial intelligence and data analytics competencies for public health professionals. J Public Health Policy.

[6] (2024). Defining essential public health functions and services to strengthen national workforce capacity. https://www.who.int/publications/i/item/9789240091436.

[7] Frank JR, Snell LS, Cate OT (2010). Competency-based medical education: theory to practice. Med Teach.

[8] The Council on Linkages Between Academia and Public Health Practice (2021). Core competencies for public health professionals. PHF.

[9] Yardley S, Dornan T (2012). Kirkpatrick’s levels and education “evidence”. Med Educ.

[10] Miao F, Holmes W, Huang R, Zhang H (2021). AI and Education: Guidance for Policy-Makers.

[11] Nagi F, Salih R, Alzubaidi M (2023). Applications of artificial intelligence (AI) in medical education: a scoping review. Stud Health Technol Inform.

[12] Chan KS, Zary N (2019). Applications and challenges of implementing artificial intelligence in medical education: integrative review. JMIR Med Educ.

[13] Pizzolla I, Aro R, Duez P, De Lièvre B, Briganti G (2023). Integrating artificial intelligence into medical education: lessons learned from a Belgian initiative. J Interact Learn Res.

[14] Uddin J, Feng C, Xu J (2025). Health communication on the internet: promoting public health and exploring disparities in the generative AI era. J Med Internet Res.

[15] Venturini P, Faria PL, Cordeiro JV (2025). AI and omics technologies in biobanking: applications and challenges for public health. Public Health.

[16] Benke K, Benke G (2018). Artificial intelligence and big data in public health. Int J Environ Res Public Health.

[17] Argentieri MA, Amin N, Nevado-Holgado AJ (2025). Integrating the environmental and genetic architectures of aging and mortality. Nat Med.

[18] Noorbakhsh-Sabet N, Zand R, Zhang Y, Abedi V (2019). Artificial intelligence transforms the future of health care. Am J Med.

[19] (2025). Experience the power of assistive technology with OrCam’s AI devices. OrCam.

[20] Jungwirth D, Haluza D (2023). Artificial intelligence and public health: an exploratory study. Int J Environ Res Public Health.

[21] İlgen B, Pilic A, Harder T, Hattab G (2024). NLPIR ’23: Proceedings of the 2023 7th International Conference on Natural Language Processing and Information Retrieval.

[22] Chen S (2025). How much energy will AI really consume? the good, the bad and the unknown. Nature.

[23] Ding Z, Wang J, Song Y (2025). Tracking the carbon footprint of global generative artificial intelligence. Innovation (Camb).

[24] Wolf RM, Abramoff MD, Channa R, Tava C, Clarida W, Lehmann HP (2022). Potential reduction in healthcare carbon footprint by autonomous artificial intelligence. NPJ Digit Med.

[25] Miao F, Holmes W (2023). Guidance for Generative AI in Education and Research.

[26] Abdulnour REE, Gin B, Boscardin CK (2025). Educational strategies for clinical supervision of artificial intelligence use. N Engl J Med.

[27] Char DS, Shah NH, Magnus D (2018). Implementing machine learning in health care - addressing ethical challenges. N Engl J Med.

[28] Obermeyer Z, Powers B, Vogeli C, Mullainathan S (2019). Dissecting racial bias in an algorithm used to manage the health of populations. Science.

[29] (2021). Ethics and governance of artificial intelligence for health: WHO guidance. https://www.who.int/publications/i/item/9789240029200.

[30] (2025). Ethics and governance of artificial intelligence for health: guidance on large multi-modal models. https://www.who.int/publications/i/item/9789240084759.

[31] (2025). Shaping ethics, regulation and standardization in AI for health - ITU-WHO Focus Group on AI for Health. http://handle.itu.int/11.1002/pub/82693bf5-en.

[32] European Union (2024). Regulation (EU) 2024/1689 of the European Parliament and of the Council of 13 June 2024 laying down harmonised rules on artificial intelligence and amending Regulations (EC) no 300/2008, (EU) no 167/2013, (EU) no 168/2013, (EU) 2018/858, (EU) 2018/1139 and (EU) 2019/2144 and directives 2014/90/EU, (EU) 2016/797 and (EU) 2020/1828 (artificial intelligence act) (text with EEA relevance). EUR-Lex.

[33] Italian Republic (2025). Disposizioni e deleghe al Governo in materia di intelligenza artificiale. (25G00143) (GU Serie Generale n.223 del 25-09-2025) [Article in Italian]. Gazzetta Ufficiale.

[34] Rajpurkar P, Chen E, Banerjee O, Topol EJ (2022). AI in health and medicine. Nat Med.

[35] Arora A (2023). Moravec’s paradox and the fear of job automation in health care. Lancet.

[36] Friedman CP (2009). A “fundamental theorem” of biomedical informatics. J Am Med Inform Assoc.

[37] Wang X, He X, Wei J, Liu J, Li Y, Liu X (2023). Application of artificial intelligence to the public health education. Front Public Health.

